# The cardiac phospho-proteome during pressure overload in mice

**DOI:** 10.1038/s41597-025-05506-7

**Published:** 2025-08-02

**Authors:** Rhys Wardman, Steve Grein, Jennifer Schwartz, Frank Stein, Joerg Heineke

**Affiliations:** 1 Department of Cardiovascular Physiology, European Center for Angioscience (ECAS), Medical Faculty Mannheim of Heidelberg University, Mannheim, Germany; 2 German Center for Cardiovascular Research (DZHK), partner site Heidelberg/Mannheim, Mannheim, Germany; 3 Proteomics Core Facility, European Molecular Biology Laboratory (EMBL), Heidelberg, Germany; 4 Present Address: Department of Internal Medicine III (Cardiology, Angiology, and Pneumology), Heidelberg University Hospital, Heidelberg, Germany

**Keywords:** Cardiac hypertrophy, Proteomics

## Abstract

Transaortic constriction (TAC) is a murine model of pressure overload-induced cardiac hypertrophy and heart failure. Despite its high prevalence during aortic stenosis or chronic arterial hypertension, the global alterations in cardiac phospho-proteome dynamics following TAC remain incompletely characterised. We present a database of the phospho-proteomic signature one day and seven days after TAC. Utilising proteomic and phospho-proteomic analyses, we quantified thousands of proteins and phosphorylation sites, revealing hundreds of differential phosphorylation events significantly altered in the cardiac response to pressure overload. Our analysis highlights significant changes in hypertrophic signalling, metabolic remodelling, contractile function, and the stress response pathways. We present proteomic data from the main cardiac cell types (endothelial cells, fibroblasts and cardiomyocytes) to reveal the cellular localisation of the detected phospho-proteins, offering insights into temporal and site-specific phosphorylation events, facilitating the potential discovery of novel therapeutic targets and biomarkers. By making this resource publicly available (ProteomeXchange with identifier PXD061784) we aim to enable further exploration of the molecular basis of cardiac remodelling and advance translational research in heart failure.

## Background & Summary

The process of pathological cardiac remodelling is highly complex and occurs in response to various mechanical, metabolic and molecular stressors, for example during myocardial infarction, diabetic cardiomyopathy or aortic valve stenosis. At the macroscopic level, it often entails an increase in cardiac size and changes in ventricular shape and thickness of ventricular walls^1–3^. Microscopically, remodelling during disease is associated with cardiomyocyte growth (termed hypertrophy), cardiac fibroblast proliferation and exaggerated deposition of extracellular matrix as well as increased adaptive angiogenesis in the short term. Pathological remodelling ultimately leads to the development of heart failure, driven by a number of cellular mechanisms such as increasing cardiomyocyte lengthening, contractile dysfunction and cell death, fibrosis, changing metabolism and emerging capillary rarefaction^3–5^. These changes themselves are driven by numerous transcriptional and post-transcriptional reprogramming events including the activation of stress signalling pathways, which in turn drive alterations in the expression and function of the cardiac proteome^4,6,7^.

Changes in protein phosphorylation, downstream of specific signalling cascades mediating changes in contractile protein function (e.g. TTN and Myl2 phosphorylation), hypertrophic signalling (e.g. CaMKII, MAPK pathways), metabolic circuits (e.g. AMPK phosphorylation), fibrotic signalling (e.g. TGF-β mediated SMAD phosphorylation) and electrophysiological properties (e.g. RyR2 phosphorylation) are key temporal regulators of adaptive and maladaptive cardiac responses and the transition towards heart failure^8–10^. Therefore, investigating the global changes in phosphorylation patterns of cardiac proteins and the cell type it mainly occurs in, as well as identifying so far unexplored or unknown phosphorylation events could provide invaluable insights into the post-translational mechanisms underlying pathological heart remodelling.

Here, we make available a phospho-/proteomic dataset of global changes in cardiac protein phosphorylation occurring acutely one day, or sub-acutely seven days after the induction of transaortic constriction (TAC) in mice. TAC, an established and commonly used surgical model of pressure overload, results in distinct phases of cardiac remodelling, hypertrophy and progressively deceased heart function as it occurs in patients suffering from arterial hypertension or aortic stenosis^11–13^.

In order to identify the temporal dynamics underlying the acute phase, accounting for the early stress responses, early growth factor signalling and altered contractility, and the following subacute remodelling phase, accounting for cardiomyocyte hypertrophy, cytoskeletal and structural remodelling, metabolic reprogramming, angiogenesis and fibrosis initiation, we identified the global changes in phosphorylation occurring in the heart one day or seven days following TAC. The acute response to TAC, as we have defined here as one-day after surgery, has been shown to exhibit distinct changes in metabolism, cardiomyocyte function and inflammation, representing the initiation of multiple signalling programmes directly preceding detectable cardiac hypertrophy^14–17^. On the other hand, the subacute phase, as we defined here as seven days after surgery, represents a period of enhanced extracellular matrix deposition, increased cardiomyocyte size, and concentric hypertrophy, in a stage preceding the development of systolic dysfunction and ultimate heart failure^13,14^.

Furthermore, we have included data on the cell type specific expression patterns of differentially phosphorylated proteins within the heart. We hope that these data sets can serve as a valuable resource for further exploration into the post-translational mechanisms underlying cardiac remodelling and heart failure.

## Methods

### Mice and experimental conditions

10-week-old C57BL/6 N mice were used for both 1-day and 1-week TAC/Sham experiments.

The animal experiment protocols were approved under animal license number G-228/18.

### TAC/Sham surgical procedure

Mice were anaesthetised with 3% isoflurane in a whole-body chamber, maintained with 2% isoflurane via a face mask, with preoperative analgesia with buprenorphine (0.1 mg/kg body weight) and Rimadyl (10–15 mg/kg body weight), subcutaneously. Mice were placed on a heated surgical table (37 °C), and the ventral thorax shaved and disinfected. An incision was made from the fourth rib to the sternum, and the sternum opened by an upper median sternotomy to the level of the third rib. The thymus was separated and the aortic arch exposed. The aorta was ligated/constricted using a 7-0 silk suture around a 26-gauge needle between the carotid arteries, and the needle removed. Sham surgeries followed the same procedure without aortic ligation. Atropine sulphate (0.1 mg/kg body weight, subcutaneously) was administered, the sternum closed with interrupted sutures, and skin incision closed with additional sutures and sealed with Histoacryl tissue glue. Mice were monitored postoperatively in a 28 °C incubator until fully awake, and postoperative analgesia included buprenorphine (0.1 mg/kg body weight, subcutaneously) and metamizole (200 mg/kg body weight, via drinking water) administered as post-operative analgesia.

### Sample preparation for phospho/proteomic analysis

Mice were euthanised by cervical dislocation, the chest disinfected with 70% ethanol, and the heart excised and washed in ice-cold PBS. Hearts were dissected at the mid-ventricular level, with the basal parts of ventricle processed further. Samples were lysed in 5 volumes of lysis buffer (100 mM Tris HCl pH 8.5, 7 M Urea, 1% Triton X-100, 5 mM Tris(2-carboxyethyl)phosphinhydrochloride, 55 mM 2-Chloroacetamide, 10 Units/mL DNase I, 1 mM Magnesium chloride, 1 mM Sodium orthovanadate, 1 x protease inhibitors, 1 x Complete mini EDTA free protease inhibitor). Samples were homogenised and DNA sheered in a Bioruptor for 45 minutes (20 seconds on, 40 seconds off). Residual cell debris was removed by centrifugation and 1% Benzonase was added to the supernatant for 30 minutes at room temperature. A Bradford assay was performed and protein concentration normalised to 1 mg for each sample (in lysis buffer). To the 1 mg of protein sample, four volumes of methanol, one volume of chloroform and 3 volumes of ultrapure water were added sequentially, and samples were centrifuged at 3740 g for 15 minutes. The upper layer was removed, and three volumes of methanol added to the remaining volume, mixed, and centrifuged. The liquid phase was removed from the pellet, and the air-dried protein pellets were submitted for phospho/proteomic analysis.

### Sample preparation of purified cardiomyocytes, cardiac endothelial cells and fibroblasts for cell type specific proteomic analysis

To obtain purified cardiac cell populations for proteomic analysis, cardiomyocytes (CM), endothelial cells (EC), and fibroblasts (FB) were isolated from murine hearts using a combination of enzymatic digestion, Langendorff perfusion method (CMs), and magnetic-activated cell sorting (MACS) (ECs & FBs).

### Isolation of cardiac endothelial cells and fibroblasts

Mice were euthanised and hearts collected as before. The tissue was weighed and minced into small fragments in an enzyme solution containing 500 U/mL Collagenase I (Worthington, LS004176) and 150 U/mL DNAse I in RPMI medium and digestion at 37 °C for 30 minutes. The resulting cell suspension was filtered through a 70 µm cell strainer and washed with fetal calf serum (FCS) and MACS buffer.

For endothelial cell isolation, the pellet was resuspended in MACS buffer, incubated with CD146 microbeads (Miltenyi Biotec, 130-092-007) (20 µL per sample), and subjected to MACS separation using MS columns (Miltenyi Biotec, MS columns, 130-042-201) on a magnetic stand. Bound endothelial cells were eluted and pelleted by centrifugation at 400 × g for 6 minutes at 4 °C, then processed for proteomic analysis.

Fibroblasts were collected from the MACS flow-through fraction. The cell suspension was incubated with fibroblast feeder removal beads (Miltenyi Biotech, 130-095-531) (20 µL per sample) for 30 minutes at room temperature, followed by an additional MACS purification step. The fibroblast fraction was subsequently pelleted, resuspended in MACS buffer, and processed for proteomic analysis.

### Langendorff isolation of adult murine cardiomyocytes

Cardiomyocytes were isolated via the Langendorff perfusion method. Excised hearts were immediately placed in perfusion buffer (10 mM BDM, 55 mM Glucose in H_2_O). The aorta was cannulated and secured with a silk suture. Retrograde perfusion was initiated at a rate of 3 mL/min to clear residual blood. An enzyme solution containing Liberase DH (5 mg/mL), trypsin (1%), and CaCl₂ (100 mM) was perfused for 5–6 minutes at 37 °C to digest extracellular matrix proteins. The heart was released from the cannula, minced and homogenised. The resulting cell suspension was passed through a 100 µm strainer to remove tissue debris. Enzymatic digestion was stopped using 10% FCS, 12.5 µM CaCl2 in perfusion buffer. CM were pelleted by low-speed centrifugation (17 × g for 2 minutes) and gently resuspended in 5% FCS, 20 µM CaCl₂ in perfusion buffer. The suspension was allowed to sediment for 10 minutes, after which the cell pellet was collected for downstream analysis.

### Protein extraction and sample preparation for mass spectrometry

Isolated CMs, ECs, and FBs were lysed in a buffer containing 10 mM Tris, 150 mM NaCl, 4% glycerol, 0.5 mM sodium disulfite, 1% Triton X-100, 0.1% sodium deoxycholate, 0.05% SDS, 1 mM DTT, and protease inhibitors. Cell lysates were subjected to shock-freezing in liquid nitrogen and later thawed on ice. Following centrifugation at 12,000 × g for 10 minutes at 4 °C, the soluble protein fraction was collected.

### Mass spectrometry

#### Sample preparation

Pellets for whole proteome and phospho-proteomic analysis were resuspended in digestion buffer (100 mM Tris-HCl pH 8.5, 1% sodium deoxycholate, 5 mM Tris(2-carboxyethyl)phosphin-hydrochlorid and 30 mM chloroacetamide), trypsin was added (1:50 (w/w)) and proteins digested overnight at room temperature (RT). Digestion was stopped with 1% TFA, sodium deoxycholate was precipitated and samples were centrifuged for 10 min at 17000 g. Desalting was performed by using 30 uM Oasis HLB 96-well plates, buffer A was 0.1% formic acid and buffer B 80% acetonitrile with 0.1% formic acid. Pooled fractions were dried under vacuum centrifugation and reconstituted in 10 μL 1% formic acid, 4% acetonitrile and stored at −80 °C. Phosphopeptide enrichment was done as in Leutert *et al*.^18^. Peptides were taken up in IMAC loading solvent (80% acetonitrile, 0.4% TFA). An aliquot was used for full proteome analysis, remaining peptides were subjected to phosphopeptide enrichment using the KingFisher Apex TM platform (ThermoFisher) and magnetic Fe-NTA beads (Cube Biotech). Phosphopeptides were eluted with 0.2% dimethylamine in 80% acetonitrile. Peptides were labelled with TMT16plex and fractionated by high-pH reversed-phase fractionation^19^.

For cell specific proteomic analysis, reduction of disulfide bonds was performed with dithiothreitol (56 °C, 30 min, 10 mM in 50 mM HEPES, pH 8.5), followed by alkylation with 20 mM 2-chloroacetamide (RT, 30 min). Samples were subjected to the SP3 protocol and peptides were eluted by tryptic digestion overnight at 37 °C^20^. Peptides were recovered twice in HEPES buffer on a magnet and labelled with TMT6plex or TMT16plexaccording the manufacturer’s instructions^19,21^. Samples were combined and desalted using an OASIS® HLB µElution Plate (Waters). Pooled samples were subjected to high-pH reverse phase fractionation on an Agilent 1200 Infinity high-performance liquid chromatography system equipped with a Gemini C18 column (3 μm, 110 Å, 100 × 1.0 mm, Phenomenex) with a Gemini C18, 4 × 2.0 mm SecurityGuard cartridge as a guard column. 48 fractions were collected and pooled into 12 fractions and dried down.

### Measurement

Samples for cell type specific proteomics were measured on a Q Exactive™ Mass Spectrometer (Thermo) and whole proteome and phospho-proteomic analysis in an Orbitrap Fusion™ Lumos™ Tribrid™ Mass Spectrometer (Thermo) coupled to an UltiMate 3000 RSLC nano LC system (Dionex). Sample was concentrated on a C18 µ-Precolumn (Acclaim PepMap 100, 5 µm, 300 µm i.d. × 5 mm, 100 Å) and resolved on a nanoEase™ M/Z HSS T3 column (75 µm × 250 mm C18, 1.8 µm, 100 Å). Trapping was carried out with 30 µL/min 0.5% trifluoroacetic acid for 4 minutes. Peptides were eluted via the analytical column (solvent A: 0.1% formic acid in water, 3% DMSO) with a constant flow of 0.3 µL/min, with increasing percentage of solvent B (0.1% formic acid in acetonitrile, 3% DMSO) from 2% to 8% in 6 min, 8% to 28% in 66 min, from 28% to 40% in 10 min, followed by an increase of B from 40–80% for 3 min and a re-equilibration back to 2% B for 5 min.

For the phospho-proteomic samples the percentage of solvent B was increased as follows: from 2% to 4% in 4 min, to 8% in 2 min, to 25% in 64 min, to 40% in 12 min, to 80% in 4 min, followed by re-equilibration back to 2% B in 4 min, for the full proteome analysis, the steps were from 2% to 8% in 4 min, to 28% in 104 min, to 40% in 4 min, to 80% in 4 min, followed by re-equilibration back to 2% B in 4 min. Peptides were introduced into the mass spectrometer via a Pico-Tip Emitter 360 µm OD × 20 µm ID; 10 µm tip (New Objective) and an applied spray voltage of 2.4 kV. The capillary temperature was at 275 °C. Settings for the Q Exactive were: Full mass scan (MS1) with mass range 375–1200 m/z, profile mode, in the orbitrap, resolution of 70000, fill time 10 ms. AGC target 3E6. Data dependent acquisition (DDA) was performed with the resolution set to 17500, fill time 50 ms, AGC target of 9E1 ions with a normalised collision energy of 32, HCD, fixed first mass 110 m/z.

Settings for the Fusion Lumos were: full mass scan with mass range 375 to1400 m/z for the phosphoproteome (375 to 1500 m/z for the full proteome), in profile mode in the orbitrap with resolution of 120000. The filling time was set at maximum of 50 ms for the full proteome with a limitation of 4 × 105 ions. Data dependent acquisition (DDA) was performed with the resolution set to 30000, with a fill time of 110 ms for the phosphoproteome (94 ms for the full proteome) and a limitation of 1 × 105 ions. A normalised collision energy of 34 was applied. MS2 data was acquired in profile mode. Fixed first mass was set 110 m/z.

IsobarQuant with Mascot (v2.2.07) (for cell type samples) and Fragpipe v21.1 with MS Fragger v4.0 (for whole proteome and phosphoproteome) were used to process the acquired data, searched against a *Mus musculus* proteome database (IsobarQuant: UP000000589, May 2016, 59550 entries; MS Fragger: UP000000589, October 2022, 21968 entries) including common contaminants and reversed sequences^22,23^. The following modifications were included into the search parameters: Carbamidomethyl on Cysteine and TMT6/16 on lysine as fixed modifications, protein N-term acetylation, oxidation on methionine, phosphorylation on STY (phosphoproteome only) and TMT6/16 on N-termini as variable modifications. A mass error tolerance of 10 ppm (IsobarQuant) or 20 ppm for precursor ions and 0.02 Da (IsobarQuant) or 20 ppm for fragment ions was set (MSFragger). Trypsin was set as protease with a maximum of two missed cleavages, and minimum peptide length set at seven amino acids. The minimum peptide length was set to seven amino acids.

### Data analysis

For phospho-proteomics data, raw output files of FragPipe (psm.tsv for phospho data and protein.tsv files for input data) were processed using the R language (ISBN 3-900051-07-0)^24^. Peptide spectral matches (PSMs) with a phosphorylation probability greater 0.5 and proteins with at least two razor peptides were considered. Phosphorylated amino acids were marked with a * in the amino acid sequences behind the phosphorylated amino acid, labelled with a 1, 2 or 3 for the number of phosphorylation sites in the peptide and concatenated with the protein ID to create a unique ID for each phosphopeptide. Raw TMT reporter ion intensities for all matches with the same phosphopeptide ID were summed. For the input data, the reporter ion intensities were used as given in the protein.tsv output files. Phospho signals were also normalised by input abundance according to the following formula:

$${Norm}.{{Intensity}}_{{phospho},{gene},{condition}}=\frac{\frac{{{Intensity}}_{{phospho},{gene},{condition}}}{{{Intensity}}_{{input},{gene},{condition}}}}{\frac{{{median}({Intensity}}_{{phospho},{gene}})}{{{median}({Intensity}}_{{input},{gene}})}}{{median}({Intensity}}_{{phospho},{gene}})$$

Transformed summed TMT reporter ion intensities were cleaned for batch effects using the ‘removeBatchEffects’ function of the limma package and normalised using the vsn package^25,26^. Missing values were imputed with ‘knn’ method using the Msnbase package^27^. Differential expression was tested using the limma package. Phospho, normalised phospho and input data were tested separately. The replicate information was added as a factor in the design matrix given as an argument to the ‘lmFit’ function of limma, imputed values were given a weight of 0.05. Hits were defined as a false discovery rate (fdr) smaller 5% and a fold-change of at least 100% and candidates with an fdr below 20% and a fold-change of at least 50%. Clustering with all hit phospho-peptides based on the median protein abundances normalised by median of control condition was conducted to identify groups of proteins with similar patterns across conditions. The ‘kmeans’ method was employed, using Euclidean distance as the distance metric and ‘ward.D2’ linkage for hierarchical clustering. The optimal number of clusters (3) was determined using the Elbow method, which identifies the point where the within-group sum of squares stabilizes. Gene ontology (GO) enrichment analysis (Molecular Function (MF), and Biological Process (BP)) was performed using the ‘clusterProfiler’ package using ‘org.Mm.eg.db’ as the reference database^28^. The odds ratio (‘odds_ratio’) for each GO term was calculated by comparing the proportion of genes associated with that term in the dataset (‘GeneRatio’) to the proportion in the background set (‘BgRatio’). An odds ratio greater than one indicates an enriched GO term.

For cell type specific proteomic data analysis, initial data processing included filtering out contaminants and reverse proteins. Only proteins quantified with at least two unique peptides (with qupm >= 2) were considered for further analysis. Additionally, only proteins identified and quantified in at least two out of three mass spec runs were retained to ensure robustness. 4435 proteins passed the quality control filters. Batch effects were removed using the ‘removeBatchEffect’ function of the limma package on the log2 transformed raw TMT reporter ion intensities (‘signal_sum’ columns)^25^. Normalisation was performed using the ‘normalizeVSN’ function of the limma package^26^. Missing values were imputed with the ‘knn’ method using the ‘impute’ function from the Msnbase package ensuring that incomplete data did not distort the analysis^27^. Differential expression analysis was performed using a moderated t-test accounting for replicate information by including it as a factor in the design matrix passed to the ‘lmFit’ function^25^. Imputed values were assigned a weight of 0.01, while quantified values were given a weight of one, ensuring analysis reflected the uncertainty in imputed data. Proteins were annotated as hits if they had a false discovery rate (FDR) below 0.05 and an absolute fold change greater than two. Proteins were considered candidates if they had an FDR below 0.2 and an absolute fold change greater than 1.5. Clustering with all hit and candidate proteins based on the median protein abundances normalised by median of control condition was conducted to identify groups of proteins with similar patterns across conditions. The ‘kmeans’ method was employed, using Euclidean distance as the distance metric and ‘ward.D2’ linkage for hierarchical clustering. The optimal number of clusters (20) was determined using the Elbow method. Whilst sham and TAC operated samples were processed and analysed (and included in the deposited data set), only sham data was reported in the manuscript.

## Data Records

The mass spectrometry proteomics and phospho-proteomics data have been deposited to the ProteomeXchange Consortium via the PRIDE partner repository with the dataset identifier PXD061784 under the dataset name ‘the cardiac phospho-proteome during pressure overload in mice’^29,30^.

Within this database are two datasets P1508 refers to the cell type specific proteomics, and was searched with IsobarQuant and Mascot. The corresponding raw files carry the ‘P1508’ tag in the file name and annotates all the TMT labels to the different biological conditions and the output file from the data analysis of this dataset. P3464 refers to the phospho-proteomic analysis and was analysed with two FragPipe searches of a phospho (PP) and an input (FP for full proteome) dataset. The P3464 (FP or PP) identifier identifies all analysis and output corresponding to this dataset.

## Technical Validation

**Fig. 1:**
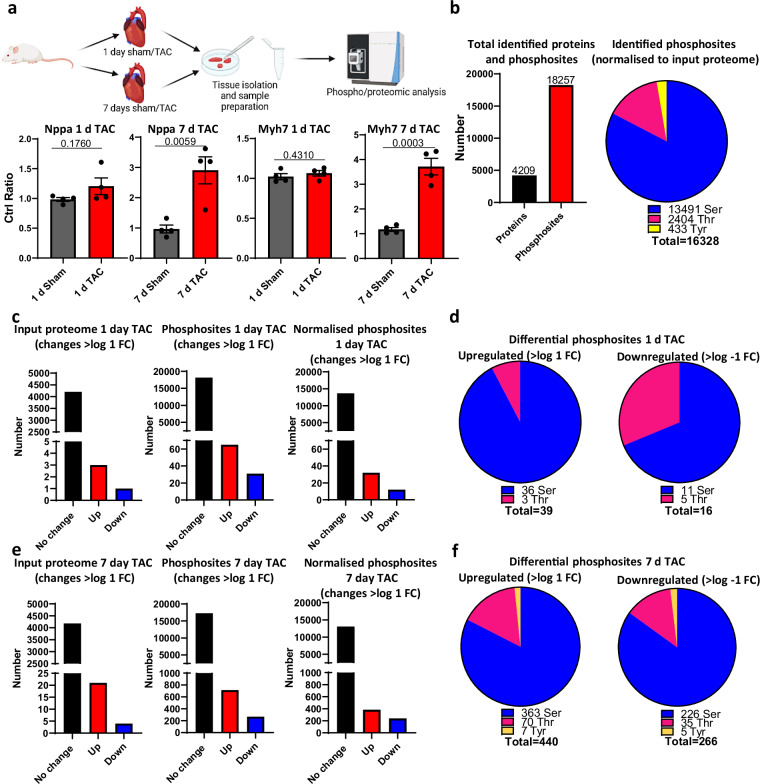
Identification of global changes in protein phosphorylation following 1 day or 7 days of transaortic constriction. (**a**) Schematic overview of experimental approach. Relative abundance of hypertrophic markers Nppa and Myh7, taken from proteomic analysis, are shown as validation of the experimental model (significance assessed by t-test, p. value indicated). (**b**) Total number of proteins (input proteome) and phosphosites identified, and breakdown of phosphosites identified after normalisation to input proteome. (**c**) Differential changes in protein (input proteome), phosphosite and normalised phosphosite abundance following 1 day of TAC (Log FC > 1). (**d**) Breakdown of differential phosphosites identified after 1 day of TAC (after normalisation to input proteome (Log FC > 1). (**e**) Differential changes in protein (input proteome), phosphosite and normalised phosphosite abundance following 7 days of TAC (Log FC > 1). (**f**) Breakdown of differential phosphosites identified after 7days of TAC (after normalisation to input proteome (Log FC > 1)).

This dataset was performed using proteomic and phospho-proteomic analysis of heart tissue in order to provide insights into the global changes in cardiac protein phosphorylation, and thereby the post-translational mechanisms underlying cardiac remodelling following one or seven days of TAC (Fig. 1a). This analysis provided coverage of 4209 proteins in the proteome, and 18257 phospho-sites within these proteins, which were predominantly serine residues (Fig. 1b). Following one day of TAC, very few changes in total protein expression were detected (three upregulated, one downregulated proteins), whereas 65 up- and 31-downregulated changes in phosphorylation, decreasing to 32 up- and 12-downregulated significant phosphorylation events after normalisation to the input proteome, were detected (Fig. 1c). While both up- and downregulated phospho-residues were both predominantly serine, there was a small increase in the proportion of threonine residues that were downregulated after one day of TAC (Fig. 1d). Conversely, seven days post-TAC revealed that 21 proteins were upregulated and four were downregulated in the input proteome, whereas 714 phospho-sites were upregulated and 268 downregulated (385 up- and 238 downregulated phospho-sites after normalisation to the input proteome) (Fig. 1e). Again, these were consistently primarily within serine residues.

**Fig. 2:**
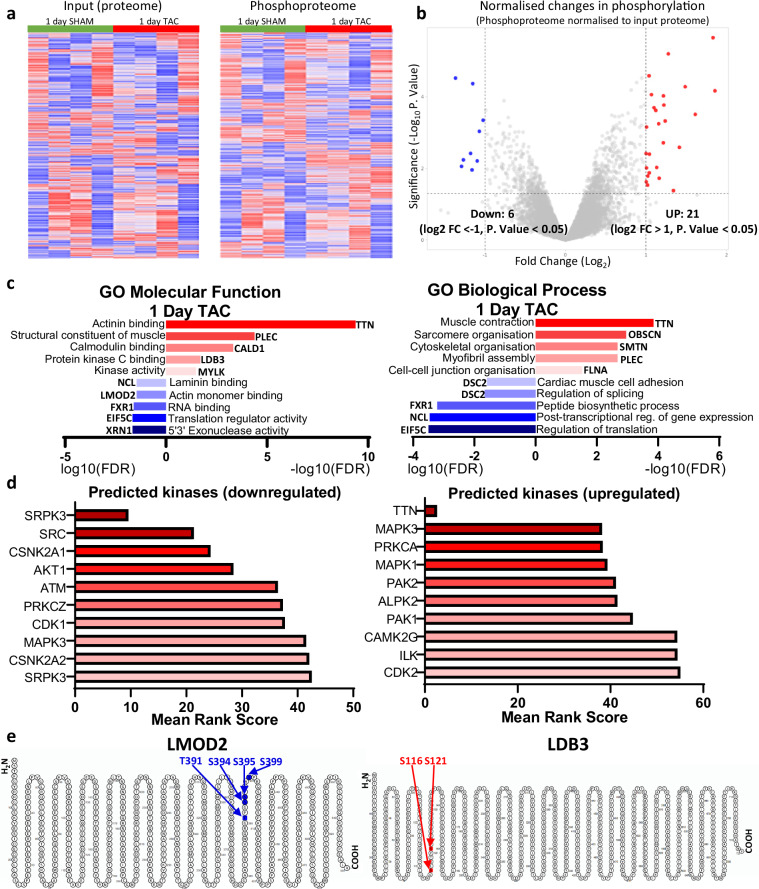
Overview of changes in phosphorylation one day post-TAC. (**a**) Heatmap of changes in protein abundance (reporter intensity) in the input proteome and in phosphosite abundance from phospho-proteomic analysis for individual replicates (n = 4) of one day sham and TAC operated mice, clustered by Spearman rank correlation. (**b**) Volcano plot showing the fold change (FC; log2) and P value (−log10) in phosphosite abundance, normalised to the input proteome, following one day of TAC vs. Sham surgery. Cut offs of differentially regulated phosphorylation of ±Log2 FC > 1 and P. Value < 0.05 are indicated. (**c**) Selected enriched GO terms (biological process, molecular function) of upregulated (red, -log10(FDR)) and downregulated (blue, log10(FDR)) phosphorylation events (taken from the ‘hits’ identified in 3b), with example protein for each term. (**d**) Mean integrated rankings of predicted kinases of upregulated and downregulated phosphorylation events (taken from the ‘hits’ identified in 3b), based on cumulative individual library rankings. (**e**) Schematic representation of differentially phosphorylated proteins after one day of TAC (significantly of ±Log2 FC > 1 and P. Value < 0.05) downregulated phosphosites highlighted in blue and upregulated phosphosites highlighted in red.

Further visualisation and filtering one-day post-TAC revealed six significant downregulated and 21 significant upregulated changes in phosphorylation (Log2 FC >± 1, P. Value < 0.05) after normalisation to the input proteome (Fig. 2a,b). Of these significant differentially phosphorylated sites, analysis of enriched Gene Ontology (GO) molecular function and biological process terms shows that a number of proteins that are already differentially phosphorylated at this early TAC time-point have functions in cardiac muscle contraction and remodelling, such as titin and obscurin, as well as signalling proteins with kinase related functions, such as myosin light chain kinase, suggesting that phosphorylation may play a very early role during this acute phase of remodelling (Fig. 2c). Interestingly, there was also a differential phosphorylation of various proteins with functions in the post-translational regulation of gene/protein expression, including proteins involved in the regulation of translation dynamics such as EIF5C and NCL. This finding aligns with recent findings that changes in expression of specific RNAs specifically at the translational level play an important role in determining early cardiac responses to haemodynamic stress following TAC, and may provide insights into the changes in phosphorylation of translation related machinery that determine these responses^31^. Analysis of the upstream predicted kinases that are linked to these differential phosphorylation sites revealed various kinases, many with known roles in heart function and contractility, likely play a role in mediating the differential phosphorylation patterns (Fig. 2d). These include a predicted upregulation of phosphorylation events by MAPK1 and MAPK3, validating this data against other studies which have shown MAPK1/3 play a role in the early cardiac response to pathological stimuli^9,32^.

As examples of differentially phosphorylated proteins at the one-day post-TAC time point, we highlighted the downregulated phosphorylation sites within LMOD2 (Leiomodin2) and upregulated phosphorylation sites within LDB3 (LIM Domain Binding 3) (Fig. 2e). LMOD2 is an important regulator of actin filament assembly in cardiac muscle tissue, essential for normal cardiomyocyte and heart function, although specific information on the phosphorylation sites that determine differential cardiac responses are sparse, demonstrating that our dataset may provide novel insights into the role of phosphorylation in determining cardiac function in response to pathophysiological challenge^33,34^. LDB3 is crucial in maintaining z-disk integrity and is associated with cardiomyopathies including following mechanical stress and, interestingly, increased LDB3 phosphorylation downstream of P38 MAPK has been suggested to play a potential role in pathological cardiac remodelling^35,36^.

**Fig. 3:**
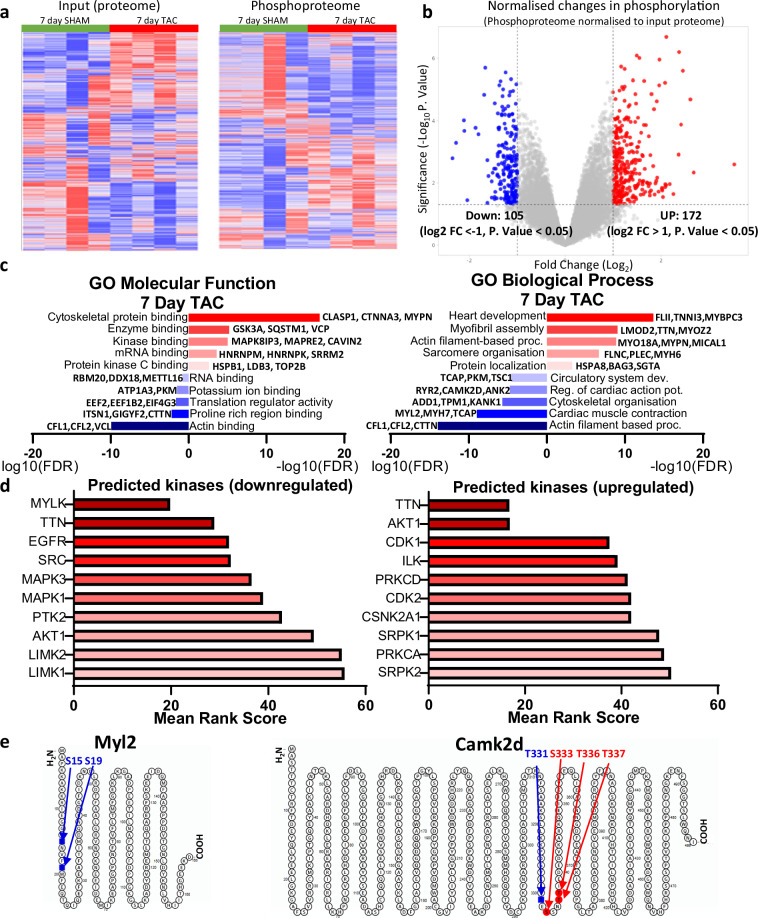
Overview of changes in phosphorylation seven days post-TAC. (**a**) Heatmap of changes in protein abundance (reporter intensity) in the input proteome and in phosphosite abundance from phospho-proteomic analysis for individual replicates (n = 4) of seven days sham and TAC operated mice, clustered by Spearman rank correlation. (**b**) Volcano plot showing the fold change (FC; log2) and P value (−log10) in phosphosite abundance, normalised to the input proteome, following seven days of TAC vs. Sham surgery. Cut offs of differentially regulated phosphorylation of ±Log2 FC > 1 and P. Value < 0.05 are indicated. (**c**) Selected enriched GO terms (biological process, molecular function) of upregulated (red, −log10(FDR)) and downregulated (blue, log10(FDR)) phosphorylation events (taken from the ‘hits’ identified in 3b) with example proteins for each term. (**d**) Mean integrated rankings of predicted kinases of upregulated and downregulated phosphorylation events (taken from the ‘hits’ identified in 3b), based on cumulative individual library rankings. (**e**) Schematic representation of differentially phosphorylated proteins after seven days of TAC (significantly of ±Log2 FC > 1 and P. Value < 0.05) downregulated phosphosites highlighted in blue and upregulated phosphosites highlighted in red.

Further analysis of the changes in phosphorylation that occur following seven days of TAC identified 105 downregulated and 172 upregulated phosphorylation events (Log2 FC >  ± 1, P. Value < 0.05) after normalisation to the input proteome, considerably more than one-day post TAC indicating the more extensive remodelling and signalling pathways activated at this time-point (Fig. 3a,b). The enriched GO terms of proteins exhibiting significant differences in phosphorylation represented a range of molecular functions and biological processes that highlight the structural rearrangements (such as actin and sarcomere organisation), cardiac development and remodelling and changes in protein dynamics (Fig. 2c). Again, there is was an enrichment of proteins with functions involved in translation regulation, aligning with findings that regulation of protein expression at the translational level plays an important role in cardiac remodelling post-TAC. At the seven-day time-point, there was a particular upregulation in the phosphorylation of proteins involved in heart development, underling the reactivation of developmental signalling and reprogramming pathways to facilitate the increased burden on the heart^37^. The predicted upstream kinases of these proteins include a number of kinases with key roles in heart remodelling, with the kinase activity of titin (TTN) predicated to be upstream of many changes, demonstrating its role in mediating mechanical stress induced changes and cardiac hypertrophy, as well as an upregulation of AKT, shown to play a role in developmental reprogramming and pathological remodelling upon pressure overload and thereby coinciding with the increased phosphorylation of proteins involved in heart development (Fig. 3d)^38,39^.

As examples of differentially phosphorylated proteins seven-days post-TAC, we visualised the differential phosphorylation sites within MYL2 (Myosin Light Chain 2), a sarcomere protein with important roles in cardiac contraction and cardiomyopathies, and CAMK2D (calcium/calmodulin-dependent protein kinase type 2 d), the major CAMK2 isoform expressed in the heart playing key roles in calcium homeostasis in cardiomyocytes and linked to the decompensation from cardiac hypertrophy to heart failure (Fig. 3d)^40–42^. Interestingly, in the case of MYL2, the dephosphorylation of Ser15, as we also identified, has been shown to directly contribute to cardiac hypertrophy and dysfunction, impaired calcium sensitivity and contractile force of cardiac muscle, whereas dephosphorylation of the Ser19 inhibits myofilament movement and contraction^40,43^. Likewise, the phosphorylation of the Ser333 and Thr366 residues of CAMK2D have been implicated in increased cardiac hypertrophy and inflammation^44^. Together, this demonstrates that differential changes in protein phosphorylation occurring seven days following TAC surgery play pivotal roles in determining pathological cardiac remodelling. It validates the pathophysiological importance of the differential phosphorylation events identified in this screen, covering changes in phosphorylation known to play roles in pathological cardiac remodelling as well as novel phosphorylation sites in this context.

**Fig. 4:**
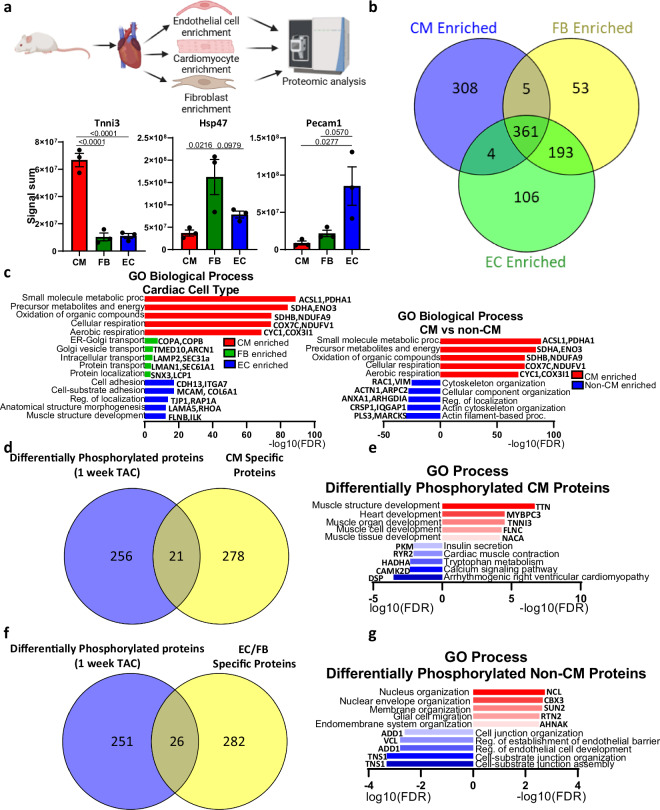
Identification of cell type specific changes in protein phosphorylation following TAC. (**a**) Cardiomyocyte (CM), endothelial (EC) and fibroblasts (FBs) were enriched from heart tissue and identified by proteomic analysis, the signal sum of selected CM, FB and EC marker proteins from proteomic analysis is shown (significance tested by one-way ANOVA). (**b**) Identification of proteins specifically enriched (Log2FC > 1, p < 0.05) in ECs, CMs or FBs. (**c**) Top five enriched GO processes (FDR) of proteins specifically enriched in ECs, FBs or CMs or in CMs vs non-CMs (EC and FB specific proteins). (**d**) Overlap of differentially phosphorylated proteins after 1 week of TAC (log2 FC >  ± 1, P. Value < 0.05) and CM specific proteins. (**e**) Top five enriched GO processes (FDR) of CM specific proteins exhibiting increased (red, −Log10(FDR)) or decreased (blue) phosphorylation after 1 week of TAC (overlap from 4d). (**f**) Overlap of differentially phosphorylated proteins after 1 week of TAC (log2 FC >  ± 1, P. Value < 0.05) and EC/FB (non-CM) specific proteins. (**g**) Top five enriched GO processes (FDR) of non-CM specific proteins exhibiting increased (red, −Log10(FDR)) or decreased (blue) phosphorylation after 1 week of TAC (overlap from 4f).

In addition to the phospho/proteomic analysis of heart tissue, we also performed proteomic analysis of the proteomes of the three major cardiac cell types in the heart, cardiomyocytes (CMs), endothelial cells (ECs) and fibroblasts (FBs), under un-operated conditions, the data sets for which are also deposited alongside this article (Fig. 4a). This identified that while 361 proteins were consistently expressed in all of the three cell types, a large number of proteins exhibited cell type specific patterns (Fig. 4b). Proteins that were exclusively enriched in CMs exhibited enrichment for GO biological processes heavily linked to cellular respiration, such as PDHA1, ENO3, COX7C and COX3L1, whereas FBs and ECs showed particular enrichment for terms linked to protein secretion/transport, such as COPA and COPB, and cell adhesion/morphogenesis, such as MCAM and ITGA7, respectively (Fig. 4c). For insights into how the differential changes in protein phosphorylation following one week of TAC occur in a cell type specific manner, we overlapped the differentially phosphorylated proteins (identified in Fig. 3) with proteins that were either specifically enriched in CMs, or specifically enriched in ECs and FBs (i.e. non-CM proteins). This found that 21 CM specific proteins were differentially phosphorylated one week post-TAC (Fig. 4d). These CM specific differentially phosphorylated proteins showed enrichment for GO biological process terms in muscle and heart development (increased phosphorylation) and muscle contraction and metabolism (decreased phosphorylation) (Fig. 4e). A total of 26 non-CM proteins (specifically enriched in ECs and/or FBs) were differentially phosphorylated one week post-TAC (Fig. 4f). These EC and FB proteins that exhibited differentially changes in phosphorylation were particularly enriched for GO terms involved in nuclear and membrane organisation (increased phosphorylation) and endothelial junction and barrier function (decreased phosphorylation) (Fig. 4g). For example, we found increased phosphorylation within S34 and S40 of nucleolin (NCL), a multifunctional RNA binding protein with functions including ribosome biogenesis, RNA metabolism, cell surface receptor and chromatin remodelling which is known to play keys roles in regulating endothelial cell function and angiogenesis^45,46^. Interestingly, the phosphorylation of NCL at both S34 and S40 residues has been shown to increase its RNA binding activity downstream of MAPK1/3 activation, resulting in increased ribosome biogenesis in the context of tumorigenesis^47^. This suggests that our data may provide novel insights into how differential changes in phosphorylation, and thereby the RNA binding, of NCL may also play a role in shaping changing in the endothelial translatome during pathological cardiac remodelling.

Together, these datasets provide detailed information into the global changes in cardiac protein phosphorylation occurring one day and seven days after TAC and therefore provide insight into the post-translational mechanisms underlying cardiac dysfunction, hypertrophy and other features of pathological remodelling (e.g. cell death, fibrosis, and metabolism). With coverage of over 4000 proteins and over 18,000 phospho-sites, we hope that this data can serve as a resource for those seeking specific information on proteins of interest in this setting and can be a platform for the further exploration of the molecular basis of cardiac remodelling and heart failure. Our initial exploration of this data, as presented here, has validated that this data represents key mechanisms underlying pathological heart remodelling, including coverage of differential phosphorylation sites with known, but also unknown role in heart disease.

Limitations to the screen include limited coverage and lack of nuclear/cytoplasmic resolution required to, for example, elucidate in further depth the role of phosphorylation on transcription factor activity within the nucleus. Whilst exhibiting a clear enrichment of certain specific cell type marker genes, the proteomic analysis of the individual cell types does not exhibit entirely exclusive expression of cell specific markers, and unfortunately does not have the same depth of coverage as the proteomic analysis performed on the whole heart samples. Similarly, whilst magnetic separation was chosen for the isolation of ECs and FBs to minimise cell stress and processing, it is possible that this selection process will introduce some perturbations in the cellular stress environment, although we expect detectable differences in the cellular proteome in this timescale to be limited. We hope, nevertheless, that this cell type specific analysis may provide further insights into the changes in phosphorylation driving differential responses in cardiomyocyte and non-cardiomyocyte populations.

## Data Availability

No new code was generated in this work.
